# Exploring the Age-Old Question: What Is the Predictive Value of EEG for Future Epilepsy in Children With Complex Febrile Seizures?

**DOI:** 10.1177/08830738231171799

**Published:** 2023-05-07

**Authors:** Purva R. Choudhari, Andrea Lowden, Alison Dolce

**Affiliations:** 1 Division of Child Neurology, Department of Pediatrics, University of Texas Southwestern, Dallas, TX, USA; 2 Departments of Pediatrics & Neurology, University of Texas Southwestern and Children's Medical Center of Dallas, Dallas, TX, USA

**Keywords:** children, electroencephalography (EEG), epilepsy, febrile seizure‌

## Abstract

Children with complex febrile seizures (CFS) have increased risk for the development of epilepsy, but varying prognostic value has been ascribed to abnormal post-CFS electroencephalograms (EEGs). We conducted a retrospective cohort study of 621 children with post-CFS EEGs and identified an association between CFS and midline-vertex discharges, which were present in 52% of the 56 EEGs with interictal epileptiform discharges. Among patients who completed at least 1 year of follow-up, 24.7% subsequently developed epilepsy. Most patients had normal EEGs but 20% had interictal epileptiform discharges. Midline-vertex discharges were seen at a similar rate in children who did not develop epilepsy (55%) and those who developed epilepsy (45%). The development of epilepsy was not associated with any interictal epileptiform discharge localization. Logistic regression modeling identified 4 predictors of future epilepsy: >3 febrile seizures in 24 hours, interictal epileptiform discharges during post-CFS EEG, family history of afebrile seizures, and age of CFS onset ≥ 3 years.

Febrile seizures (FS) are the most common seizures seen in the pediatric population, occurring in children between the ages of 6 months and 5 years.^1^ They can be categorized by duration and features as simple or complex febrile seizures. Simple febrile seizures are generalized seizures that last <15 minutes and do not recur within 24 hours. A complex febrile seizure (CFS) is defined as having 1 or more of the following features: focal onset or a seizure followed by a focal neurologic deficit, duration of 15 minutes or longer, or seizure recurrence during the same 24 hours.^1^ Although parental anxiety can be seen, in most situations, febrile seizures carry low risk for the child.^2^ An estimated 2% to 10% of children with febrile seizures subsequently develop epilepsy.^3,4^ The risk for febrile seizure recurrence is greater than the risk for future epilepsy after a simple febrile seizure, with studies demonstrating 30% to 42% risk for the first FS recurrence.^1,5^

The risk for future epilepsy tends to be higher in children with a history of CFS, particularly for children having more than 1 complex feature (such as focal neurologic signs and prolonged seizure).^6^ To stratify risk, physicians have used clinical history and diagnostic studies such as electroencephalography (EEG), neuroimaging (magnetic resonance imaging [MRI] or computed tomography [CT]), and lumbar puncture.^6^ Studies have explored the use and predictive value of EEG in children with CFS, with results varying from focal paroxysmal epileptiform discharges as a prognostic factor for future epilepsy to no prognostic value ascribed to epileptiform discharges seen after a CFS.^7–9^ Although some physicians have recommended that all children with CFS receive an EEG shortly after the CFS,^6,10^ it has been suggested that an EEG should be obtained at least 7 days after the CFS to gain a more accurate representation of risk.^7^ Risk stratification is also dependent on clinical parameters such as age at CFS and family history of seizures.^7,11^

With differing results regarding the prognostic value of EEG after CFS and varied practice recommendations about the attainment of EEG, we conducted a retrospective study to assess the predictive value of paroxysmal post-CFS EEG abnormalities for development of epilepsy with a focus on localization in association with clinical parameters as well as timing of EEG acquisition.

## Methods

**Figure 1.**
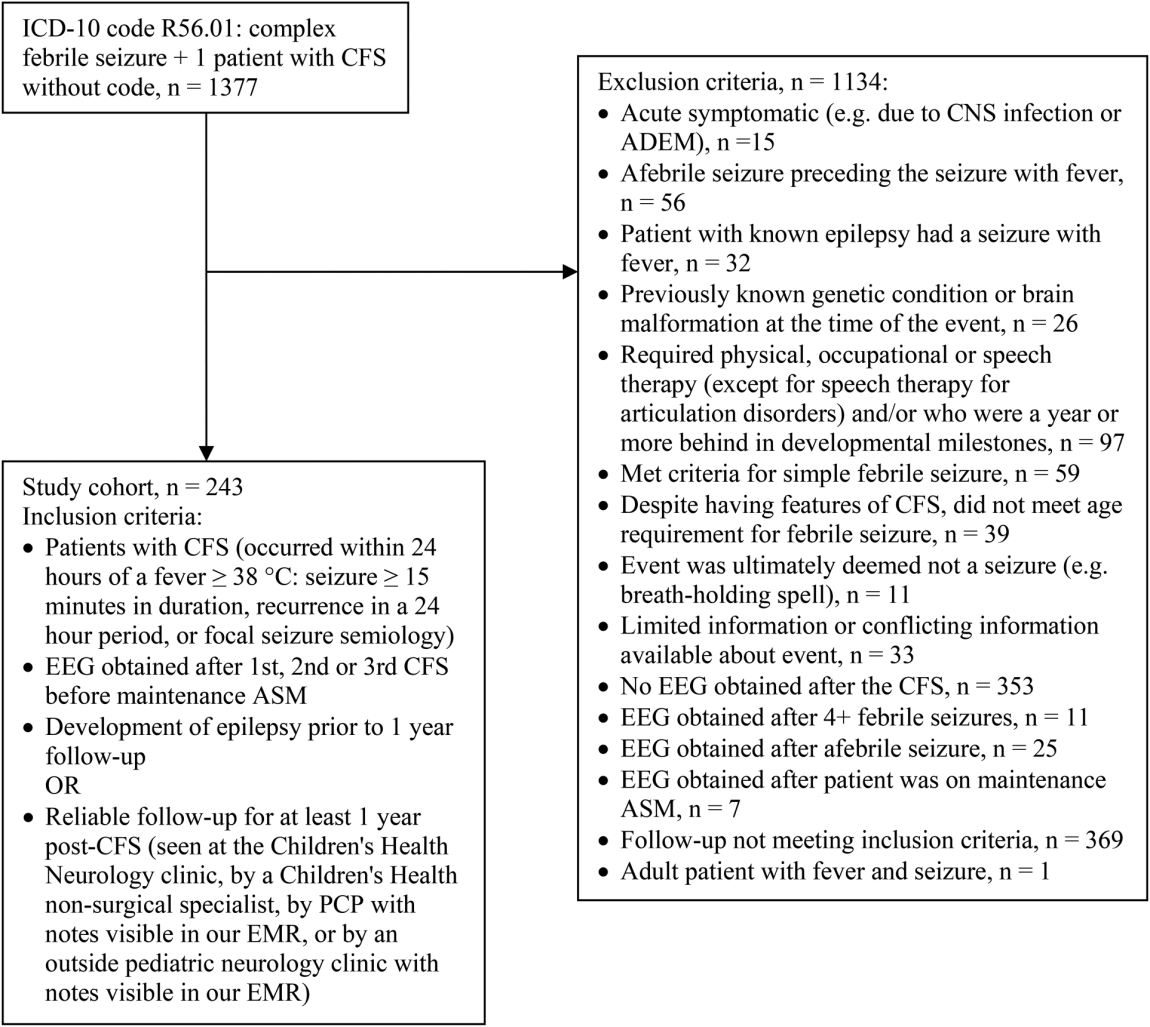
Exclusion and inclusion criteria for the study. Multiple patients were excluded for more than reason but are delineated in this figure by one exclusion criteria only. ADEM, acute demyelinating encephalomyelitis; ASM, antiseizure medication; CFS, complex febrile seizure; CNS, central nervous system; *ICD-10*, *International Classification of Diseases, Tenth Revision*.

We performed a retrospective cohort study of patients who had been seen at Children's Health Dallas between January 1, 2009, and December 31, 2019. The study was approved by the University of Texas Southwestern Medical Center Institutional Review Board. Included patients had an *International Classification of Diseases, Tenth Revision* (*ICD-10*) code for CFS (R56.01). One patient, who was diagnosed with CFS but did not have an *ICD-10* code for CFS, was added to the study. Patients had to meet the American Academy of Pediatrics criteria for CFS and have had undergone a post-CFS EEG.^1^ Patients were included in the study if they had reliable follow-up for at least 1 year or if they had developed epilepsy prior to the 1-year follow-up. Further inclusion and exclusion criteria, including those related to developmental delay and follow-up, are described in Figure 1. Henceforth, the term “index CFS” will refer to the CFS after which the EEG was obtained. The post-CFS EEG will be referred to as “index EEG.” Some patients received benzodiazepines, levetiracetam, fosphenytoin, and/or phenobarbital loads prior to the EEG to terminate seizures. The primary outcome was the development of epilepsy after CFS. Epilepsy was defined as 2 or more unprovoked seizures separated by 24 hours or by the diagnosis of an epilepsy syndrome.^12^

### EEG

Each patient's EEG was read by a board-certified pediatric neurophysiologist/epileptologist at the time of acquisition. EEG abnormalities were categorized as interictal epileptiform discharges, slowing (intermittent or continuous) and excessive beta activity. Patients were divided into one of these distinct cohorts. Interictal epileptiform discharges included sharps, spikes, polyspikes, spike and slow wave, and polyspike and slow wave discharges. Delta and theta slowing and excessive beta frequency activity (without any other abnormalities), each, were categorized as generalized or focal. Interictal epileptiform discharges were further delineated by localization: frontal, temporal, midline-vertex (Fz, Cz, or Pz), central, parietal, temporo-parieto-occipital (posterior quadrant), occipital, independent multifocal, or generalized. Interictal epileptiform discharges were considered as independent multifocal if the discharges were present in 2 or more lobes that were not contiguous and in both cerebral hemispheres. If discharge categorization was unclear from the EEG report, one of the pediatric epileptologists (principal investigator) reviewed the EEG tracing in order to categorize the discharge localization(s). Timing of EEG acquisition in relation to the patient's index CFS was distinguished as “early” for EEGs obtained within 7 days of the CFS and as “late” for EEGs obtained 7 days or greater after the CFS.

### Statistical Analysis

Descriptive statistics were obtained using Microsoft Excel and SAS OnDemand for Academics (SAS, Inc, Cary, NC). Pairwise comparisons of the proportions of multinomial categorical variables were performed using the Multinom SAS/IML module.^13^ A multivariable logistic regression analysis was performed to determine predictors of future epilepsy after CFS. Chi-squared test (Fisher exact test for n < 5) was performed to ensure no collinearities existed among the predictors used in the logistic regression model (such as duration of CFS and number of seizures during the CFS). Any patients who had been initiated on maintenance antiseizure medication after their index CFS, and did not develop epilepsy while on the antiseizure medications, were removed from analysis regarding epilepsy outcomes. Statistical significance was set at *P* < .05.

## Results

**Figure 2.**
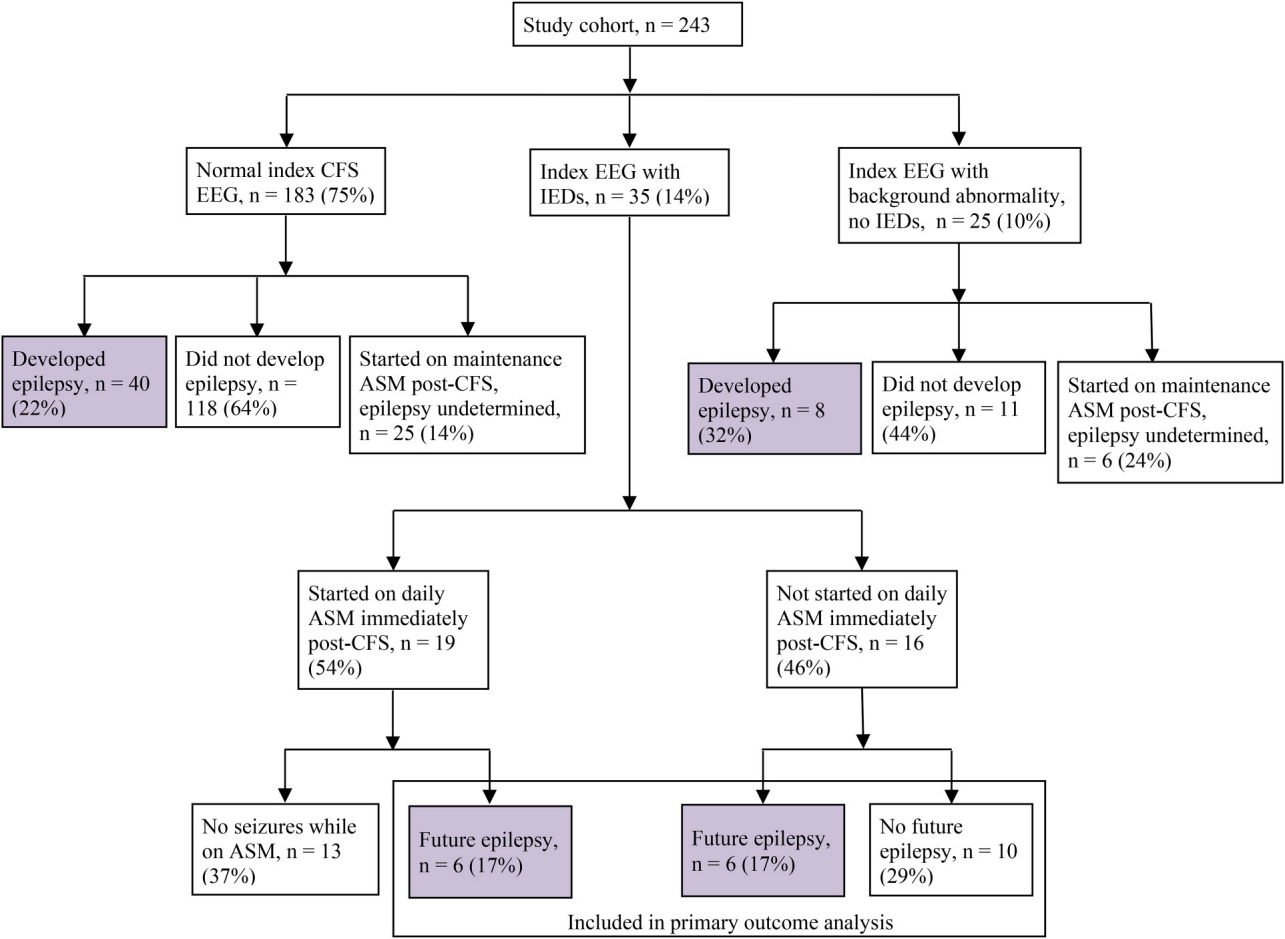
Flowchart of study cohort (patients with CFS who underwent post-CFS (index) EEG and had at least 1 year of follow-up or developed epilepsy before 1 year), the EEG characteristics, delineated as normal, IEDs, or background abnormality (generalized or focal slowing, excessive beta activity), and the numbers of patients who were started on ASMs and/or developed future epilepsy. Percentages calculated for the last row in the flowchart were obtained from the number of patients with index EEG with IEDs (n = 35). ASM, antiseizure medication; CFS, complex febrile seizure; EEG, electroencephalogram; IEDs, interictal epileptiform discharges.

Demographics and characteristics of the patients’ CFS are shown in Tables 1 and 2. Patients were followed for 14 months to 11 years, with an average follow-up time of 36 months. After their CFS, 60 patients (60/243, 24.7%) subsequently developed epilepsy. Interictal epileptiform discharges were demonstrated in 35 patients (14%, 35/243) with CFS. However, interictal epileptiform discharges were present after the index CFS for 12 of the patients (12/60, 20%) who developed epilepsy (Figure 2). After the index CFS, 50 patients (50/243, 20.6%) were started on maintenance antiseizure medication. It is worth noting that 19 of these 50 patients (38%) had interictal epileptiform discharges on the index EEG. Judging from the patients’ notes, antiseizure medications were initiated based on patient history, EEG findings, family preference, and/or physician practice preference.

**Table 1.** Demographics of Patients With Complex Febrile Seizures in the Study Cohort and Based on Development of Epilepsy.

Demographic	Total study cohort (n = 243), n (%)	Future epilepsy^a^ (n = 60), n (%)	No future epilepsy^a^ (n = 139), n (%)
Race/ethnicity			
Caucasian	66 (27.2)	26 (43.3)	27 (19.4)
African American	38 (15.6)	5 (8.3)	25 (18)
Hispanic	113 (46.5)	15 (25)	78 (56.1)
Hispanic-Black	14 (5.8)	12 (20)	2 (1.4)
Asian	3 (1.2)	0 (0)	3 (2.2)
Other	9 (3.7)	2 (3.3)	4 (2.9)
Gender			
Female	131 (53.9)	28 (46.7)	82 (59)
Male	112 (46.1)	32 (53.3)	57 (41)

^a^ Excludes patients who were started on maintenance antiseizure medications and did not develop epilepsy.

**Table 2.** Characteristics of Complex Febrile Seizures for Study Cohort and Based on Development of Epilepsy.

Characteristic	Total study cohort (n = 243), n (%)	Future epilepsy^a^ (n = 60), n (%)	No future epilepsy^a^ (n = 139), n (%)
Onset of CFS at age <3 y	229 (94.2)	54 (90)	133 (95.7)
Onset of CFS at age ≥3 y	14 (5.8)	6 (10)	6 (4.3)
Focal features	123 (50.6)	29 (48.3)	67 (48.2)
Prolonged CFS (≥15 min)	47 (19.3)	12 (20)	22 (15.8)
Febrile status (≥30 min)	39 (16)	7 (11.7)	25 (18)
≥2 seizures in 24 h	152 (62.5)	40 (66.7)	84 (60.4)
Previous febrile seizures	65 (26.7)	16 (26.7)	37 (26.6)
Family history of febrile seizures	77 (31.7)	14 (23.3)	48 (34.5)
Family history of afebrile seizures	84 (34.6)	27 (45)	40 (28.8)

Abbreviation: CFS, complex febrile seizure.

^a^ Excludes patients who were started on maintenance antiseizure medications and did not develop epilepsy.

**Figure 3.**
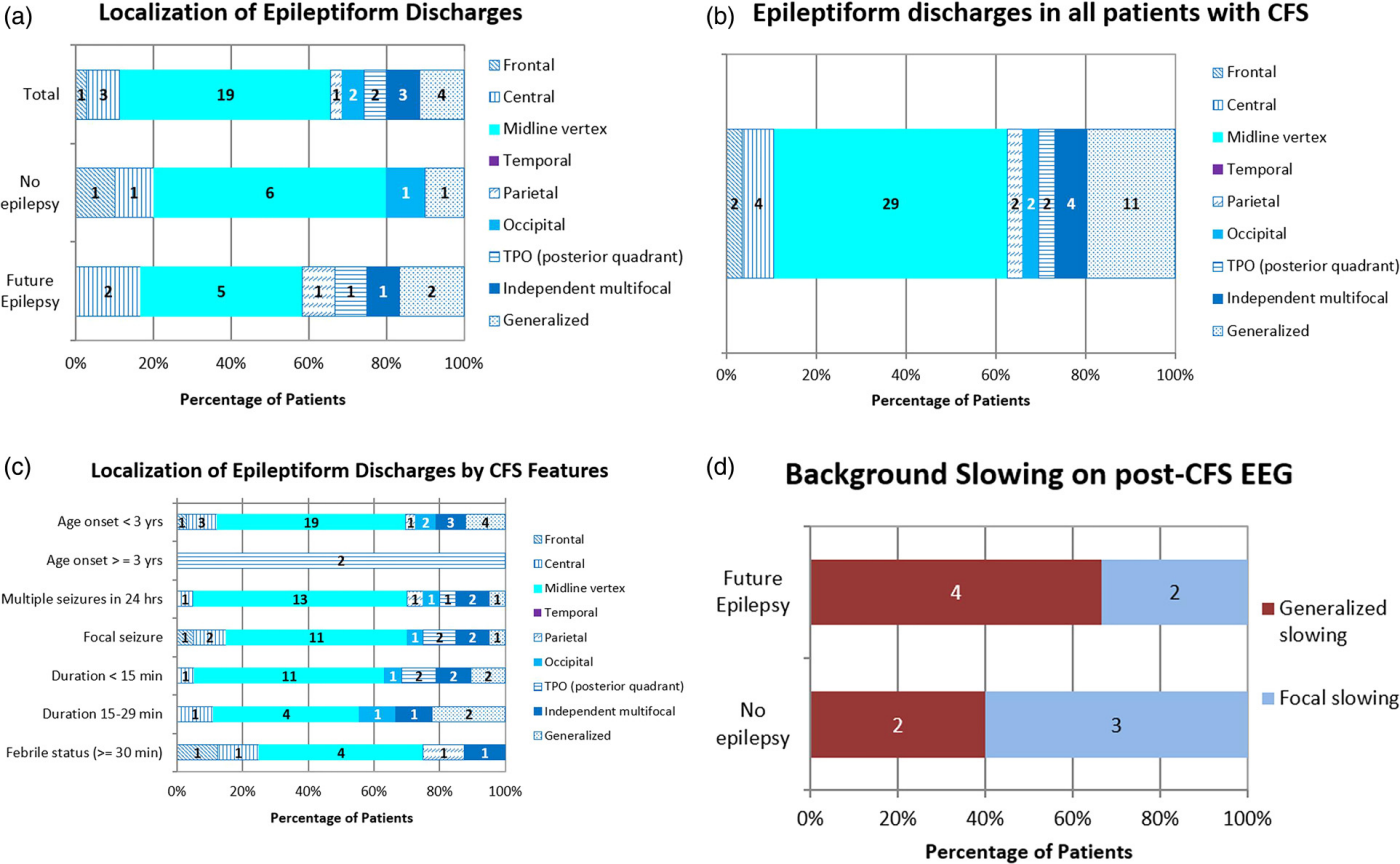
(a) Localization of IEDs in index (post-CFS) EEGs of patients included in primary outcome analysis. (b) Localization of IEDs in index (post-CFS) EEGs in all patients who underwent index (post-CFS) EEGs, regardless of follow-up. (c) Localization of IEDs on index (post-CFS) EEGs (of patients included in primary outcome analysis) delineated by CFS features. *Temporal IEDs were not seen in index (post-CFS) EEGs for any patient. (d) Background activity on index (post-CFS) EEGs of patients included in the study, separated based on those who developed future epilepsy and those who did not. CFS, complex febrile seizures; EEG, electroencephalogram; IEDs, interictal epileptiform discharges; TPO, temporo-parieto-occipital.

Midline-vertex (Fz, Cz, Pz) epileptiform discharges (19/35, 54%) were present on the index EEGs at a significantly higher rate than other epileptiform discharge localizations (*P* = .0002, when compared to generalized discharges) (Figure 3(a)). Generalized epileptiform discharges, which occurred at the next highest frequency, were present on 4 EEGs. To determine if a similar pattern held true for all patients who received a post-CFS EEG, regardless of follow-up duration, we categorized the discharge localizations seen on all EEGs with interictal epileptiform discharges (56/621) and determined that 29 (29/56, 52%) EEGs had discharges localized to the midline-vertex region. The next most frequent localization was generalized discharges, seen in 11 of 56 patients. In this group of 56 patients, significantly more patients presented with midline-vertex discharges on their index EEGs as compared to patients with generalized discharges (*P* = 0.002) (Figure 3(b)).

Children who developed epilepsy had varying localizations of interictal epileptiform discharges. No interictal epileptiform discharge localization was associated with the development of epilepsy (Figure 3(a)). Midline-vertex discharges were seen in 6 patients in the “no future” epilepsy cohort (6/10, 60%) and in 5 patients in the future epilepsy cohort (5/12, 42%), but this was not a statistically significant difference. There was no association between epileptiform discharge localizations and clinical features of CFS, such as duration of seizure, number of seizures, or focality of seizure (Figure 3(c)). Focal or generalized background slowing on EEG was seen in 6 patients (6/11, 55%) who developed epilepsy. In the cohort of patients with generalized slowing on EEG, twice as many patients developed epilepsy than those who did not develop epilepsy (Figure 3(d)).

**Figure 4.**
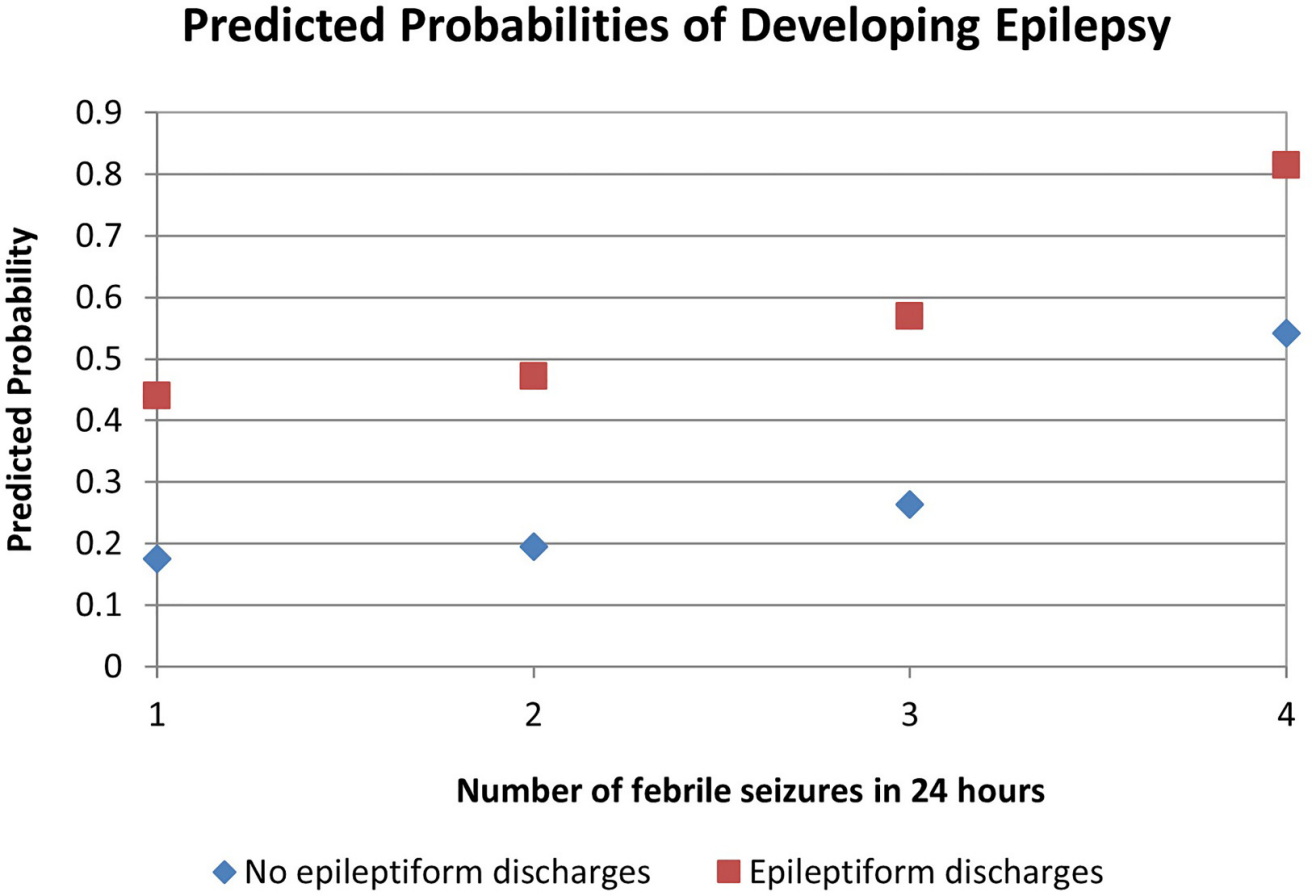
Four risk factors were identified from a multivariable logistic regression model for future epilepsy. Predicted probabilities increased if patient had a post-CFS EEG with epileptiform discharges and by number of seizures during the CFS event. This graph assumes that the patient has a family history of afebrile seizures and had CFS onset at age ≥ 3 years. CFS, complex febrile seizure; EEG, electroencephalogram.

A multivariable logistic regression analysis identified 4 risk factors as significant predictors for future epilepsy: number of febrile seizures in 24 hours, presence of interictal epileptiform discharges, family history of afebrile seizures, and age of CFS onset (Table 3 for estimated odds ratios). Five additional factors were included in the analysis but were not considered significant: history of febrile seizures, focality of CFS, seizure duration, structural abnormality on neuroimaging, and family history of febrile seizures. Based on the regression analysis, if a patient had 3 risk factors (family history of afebrile seizures, 3 years old or greater at CFS onset, and more than 3 seizures within 24 hours of the CFS event), the predicted probability of developing epilepsy was at least 50%. If the patient had all 4 risk factors (including interictal epileptiform discharges on the index EEG), the probability of developing epilepsy increased to >75% (Figure 4). Of note, these predicted probabilities were calculated using the estimated odds ratios but did not include the confidence intervals (Table 3).

**Table 3.** Odds Ratio Estimates, Confidence Intervals, and χ^2^*P* Values for Predictors of Future Epilepsy, Obtained From the Multivariable Logistic Regression Model.

Variable	Odds ratio estimate	95% confidence interval	χ^2^*P* value
>3 seizures during a 24-hour period	5.57	1.77-19.16	.0042
Age of onset of CFS ≥ 3 years	5.168	1.12-27.88	.038
Interictal epileptiform discharges present on EEG	3.704	1.37-10.43	.011
Family history of afebrile seizures	2.431	1.17-5.15	.019

Abbreviation: EEG, electroencephalogram.

Most EEGs (173/243, 71%) were obtained within 7 days of the CFS (early EEG). Interictal epileptiform discharges were present in 29 (29/173, 17%) children who underwent early EEGs compared to 6 children (6/70, 9%) who underwent late EEGs. Interictal epileptiform discharges were seen in 10 children (10/29, 34%) with early EEGs and 2 children (2/6, 33%) with late EEGs who developed future epilepsy.

The majority of children (229/243, 94%) had their initial CFS before the age of 3 years. Interictal epileptiform discharges were present in 33 children (33/229, 14%) with CFS onset at age <3 years and 2 children (2/14, 14%) with CFS onset at age ≥3 years. Epilepsy occurred in 54 children (54/187, 29%) with onset of CFS when younger than 3 years and in 6 children (6/12, 50%) with onset of CFS at or after 3 years of age. Of note, these subcohorts (187 children with CFS onset <3 years and 12 children with CFS onset ≥3 years) do not include children who were started on maintenance antiseizure medications and did not develop epilepsy.

Of the 122 children with febrile seizure recurrence, 63 children (63/243, 26%) had reported CFS recurrence. Febrile seizures recurred in 93 patients (93/122, 76%) with a normal EEG after the index CFS. In children with febrile seizure recurrence, 18 children (18/122, 15%) had interictal epileptiform discharges on their EEGs and 8 children (8/122, 7%) had background slowing. The differences between these 2 cohorts and children with normal index EEGs were not statistically significant.

EEGs were repeated (within 2-6 years) in 7 patients who had interictal epileptiform discharges on their initial post-CFS (index) EEG. Epilepsy was diagnosed in 4 patients prior to the subsequent EEG(s). A subsequent EEG in 1 patient revealed generalized slowing, and then this patient was diagnosed with epilepsy approximately 4.5 years after the index CFS. Within their follow-up duration of 3-4 years, 2 patients with interictal epileptiform discharges on subsequent EEGs did not develop epilepsy. One of these patients had an initial EEG with midline-vertex discharges followed by a normal EEG. The other patient underwent 3 EEGs: the initial EEG and second EEG had midline-vertex discharges and the third and last EEG had occipital discharges.

## Discussion

Complex febrile seizures carry a higher risk for the future development of epilepsy than simple febrile seizures.^6^ Our study noted that the majority of patients who developed epilepsy had a normal post-CFS EEG. However, of the children with a post-CFS EEG with interictal epileptiform discharges, a clinically significant 54% of these children developed epilepsy. Additionally, we identified an association between midline-vertex epileptiform discharges and CFS with equivocal predictive value for development of epilepsy. We highlighted clinical parameters that can play significant roles in the risk for future epilepsy.

Our study showed that 20% of patients who developed epilepsy had interictal epileptiform discharges on their post-CFS EEG. This is similar to previous studies in which the reported rates of epilepsy development were 25% to 33% of patients with interictal epileptiform discharges on a post-CFS EEG.^14–16^ However, Yucel et al^7^ reported that a much higher percentage (67%) of their patients with interictal epileptiform discharges on EEGs developed epilepsy. This may be related to differing selection criteria, including patients with varying degrees of developmental delays. Developmental delay has been shown to be a risk factor for the development of epilepsy after CFS.^17^ Our study excluded patients with significant delays and those who required therapies to reduce confounding the risk of epilepsy development.

Our study demonstrated that febrile seizures can be associated with midline-vertex (Fz, Cz, Pz) epileptiform discharges. To our knowledge, this specific association has not been reported in other studies. Interestingly, a 2013 retrospective study, comparing patients who had simple and complex febrile seizures and abnormal EEGs with a control group with normal EEGs, demonstrated that 56% of patients with interictal epileptiform discharges on post-CFS EEG had central discharges.^16^ However, the authors considered discharges at Cz as a central discharge along with discharges at C3 and C4,^16^ creating overlap in regions compared to the localization categories we used in our study. No study analyzing epileptiform discharge localizations considered the midline-vertex region as a separate region.^8,14,18^

Despite the association of midline-vertex discharges with CFS in our study, the presence of midline-vertex discharges do not necessarily predict epilepsy. We noted that similar numbers of patients with midline-vertex discharges on post-CFS EEGs developed and did not develop epilepsy. Our study did not find a statistically significant association between any specific epileptiform discharge localization and future epilepsy. Limited studies have investigated the correlation of interictal epileptiform discharge localization with the development of epilepsy, and the results have been variable.^8,14,16,18,19^ Three of these studies suggested that the presence of frontal discharges was a predictor for subsequent epilepsy.^8,14,18^ Our study had one patient with frontal discharges on the post-CFS EEG so we are unable to support or reject this previous finding.

Patients who had abnormal background activity without interictal epileptiform discharges were analyzed separately in our study to investigate if any prognostic value could be attributed to background slowing. Although our results indicated that slowing was not significantly associated with patients who developed epilepsy, more patients who developed epilepsy had generalized slowing (albeit the sample size was small). However, our results could be more of a marker of acute dysfunction after febrile seizures because all incidents of slowing were seen on EEGs obtained less than 7 days after the CFS.^20^ Another study evaluating a similar question reported no differences in the presence of slowing on EEGs between the cohort of patients with CFS who developed epilepsy and the cohort that did not develop epilepsy.^9^

A patient's clinical characteristics, in addition to EEG findings, contribute toward anticipated risk for epilepsy after a CFS.^6^ The age of onset of CFS has previously been suggested as a possible risk factor for the presence of interictal epileptiform discharges and a risk factor for the development of epilepsy.^7,11,16^ Our study demonstrated a similar rate of interictal epileptiform discharges regardless of onset of CFS before or after age 3 years, unlike previous studies.^15,16^ In contrast, a higher percentage of our patients with CFS onset at age ≥3 years developed epilepsy than those who had CFS onset at age <3 years, similar to Pavlidou et al.^11^

Additional clinical risk factors for epilepsy have included multiple seizures in 24 hours, prolonged seizure, focality of seizure onset, and family history of epilepsy. Yet, the significance of these risk factors varies with each study.^11,14,17^ We, like Kuang et al^14^ and Lee et al,^17^ did not identify focality as a prognostic factor based on our multivariable regression model, despite the converse finding in other studies.^11,15,19^ Similar to Lee et al^17^ and Kim et al,^15^ we found that seizure multiplicity within 24 hours of the CFS increased the risk for epilepsy. Family history of epilepsy has been frequently shown to be a predictor of epilepsy, which is concordant with our results.^11,14,19^ In fact, with a multivariable logistic regression model, we showed that the probability of developing epilepsy increased over 75% if all 4 risk factors were present (age ≥3 years at CFS onset, positive family history of afebrile seizures, multiplicity of seizures within 24 hours, and interictal epileptiform discharges on post-CFS EEG). Our data indicate that a post-CFS EEG with interictal epileptiform discharges, considered within clinical context with other clinical predictors, can have predictive value for future epilepsy.

We sought to determine if the timing of post-CFS EEG acquisition affected the nature of abnormalities seen or the rate of future epilepsy. Previous studies have generated discussions regarding the optimal timing of EEG acquisition in order to more accurately guide management.^7,22^ Our findings revealed that a higher percentage of early EEGs demonstrated interictal epileptiform discharges than late EEGs. However, the number of late EEGs performed was quite small, limiting our ability to extrapolate any significance of these findings. Some studies reported a limited presence of interictal epileptiform discharges (10% or less) on EEGs obtained within 6 days of the CFS.^7,21,23^ However, our results do not fully support the concept that detection of interictal epileptiform discharges is limited in early EEGs^22,24^ because we identified the presence of interictal epileptiform discharges in 17% of early EEGs. Similarly, Karimzadeh et al noted that 27.6% of EEGs, obtained within 48 to 72 hours post-CFS, revealed sharp waves.^22^ Yucel et al^7^ reported higher rates of future epilepsy in patients with late EEGs with interictal epileptiform discharges as compared to early EEGs, but our results indicated no difference in epilepsy development in cohorts with early versus late EEGs. The implication of the presence of interictal epileptiform discharges on early EEGs remains uncertain. We sought to determine if repeated EEGs with interictal epileptiform discharges were associated with development of future epilepsy, but there was insufficient data to conclude an association.

This study was limited by its retrospective nature and the small sample size, after stratification. Small sample size was a limitation for the analysis of timing of EEG acquisition as most of our patients underwent early EEGs. At our institution, because of practice preferences, EEGs are often obtained in hospitalized children after CFS, particularly within a short time frame of the CFS. This practice is another source of potential bias. Possible bias could have been introduced because 21 patients with interictal epileptiform discharges on post-CFS EEGs did not follow-up with our neurology clinic, and so their outcomes are unknown. Although the average follow-up time was 3 years, it is possible that some patients who eventually developed epilepsy were not captured in our data.

## Conclusions

Early identification of children who will develop epilepsy after a CFS is essential to future management and counseling for parents/caregivers. In our study, 24.7% of children presenting with CFS developed subsequent epilepsy. Four predictors of future epilepsy were identified: >3 febrile seizures in 24 hours, interictal epileptiform discharges on post-CFS EEG, family history of afebrile seizures, and age of CFS onset ≥3 years. Having all 4 risk factors can increase a patient's risk of epilepsy to greater than 75%. The majority of our patients who developed epilepsy actually had normal post-CFS EEGs, and more patients with normal EEGs had febrile seizure recurrence. However, 54% of the children who had interictal epileptiform discharges on the post-CFS EEG subsequently developed epilepsy. Additionally, antiseizure medications were initiated for 20.6% of children after CFS, 38% of whom had interictal epileptiform discharges on the post-CFS EEG. Our study also highlighted that midline-vertex discharges can be found in a significant percentage of children with CFS, but this carried an equivocal risk for development of future epilepsy. We did not find a statistically significant association between any specific epileptiform discharge localization and future epilepsy. A post-CFS EEG can provide value toward future counseling. Yet, until future prospective studies are performed, the presence of epileptiform discharges, particularly midline-vertex discharges, on post-CFS EEGs should be considered on an individual basis, in the context of clinical parameters, to guide counseling and management with antiseizure medications.
